# Acute-on-chronic liver failure alters linezolid pharmacokinetics in critically ill patients with continuous hemodialysis: an observational study

**DOI:** 10.1186/s13613-023-01184-z

**Published:** 2023-09-12

**Authors:** Tjokosela Tikiso, Valentin Fuhrmann, Christina König, Dominik Jarczak, Stefanie Iwersen-Bergmann, Stefan Kluge, Sebastian G. Wicha, Jörn Grensemann

**Affiliations:** 1 Department of Clinical Pharmacy, Institute of Pharmacy, University of Hamburg, Bundesstraße 45, 20146 Hamburg, Germany; 2 Department of Intensive Care Medicine, University Medical Center Hamburg-Eppendorf, Martinistraße 52, 20246 Hamburg, Germany; 3 Department of Medicine, Hospital of the Holy Spirit, Graseggerstraße 105, 50737 Cologne, Germany; 4 Hospital Pharmacy, University Medical Center Hamburg-Eppendorf, Martinistraße 52, 20246 Hamburg, Germany; 5 Department of Legal Medicine, University Medical Center Hamburg-Eppendorf, Martinistraße 52, 20246 Hamburg, Germany

**Keywords:** Antibiotics, Target attainment, Intensive care, Volume of distribution, Monte-Carlo simulation, Population pharmacokinetics, Integrated dialysis pharmacometric model, Probability of target attainment

## Abstract

**Background:**

In acute-on-chronic liver failure (ACLF), adequate antibiotic dosing is challenging due to changes of drug distribution and elimination. We studied the pharmacokinetics of linezolid in critically ill patients with ACLF during continuous renal replacement therapy compared to patients without concomitant liver failure (NLF).

**Methods:**

In this prospective cohort study, patients received linezolid 600 mg bid. Linezolid serum samples were analyzed by high-performance liquid chromatography. Population pharmacokinetic modelling was performed followed by Monte-Carlo simulations of 150 mg bid, 300 mg bid, 450 mg bid, 600 mg bid, and 900 mg bid to assess trough concentration target attainment of 2–7 mg/L.

**Results:**

Eighteen patients were included in this study with nine suffering from ACLF. Linezolid body clearance was lower in the ACLF group with mean (standard deviation) 1.54 (0.52) L/h versus 6.26 (2.43) L/h for NLF, P < 0.001. A trough concentration of 2–7 mg/L was reached with the standard dose of 600 mg bid in the NLF group in 47%, with 42% being underexposed and 11% overexposed versus 20% in the ACLF group with 77% overexposed and 3% underexposed. The highest probability of target exposure was attained with 600 mg bid in the NLF group and 150 mg bid in the ACLF group with 53%.

**Conclusion:**

Linezolid body clearance in ACLF was markedly lower than in NLF. Given the overall high variability, therapeutic drug monitoring (TDM) with dose adjustments seems required to optimize target attainment. Until TDM results are available, a dose reduction may be considered in ACLF patients to prevent overexposure.

**Supplementary Information:**

The online version contains supplementary material available at 10.1186/s13613-023-01184-z.

## Background

Critically ill patients with acute-on-chronic liver failure (ACLF) are at risk of acquiring infections with consecutive sepsis and septic shock, which are associated with a high mortality [1, 2]. Early empiric broad-spectrum antibiotic therapy is required to reduce mortality [3]. Linezolid is often used to cover the gram-positive spectrum, particularly in the presence of vancomycin-resistant enterococci. Acute kidney injury is the most frequent type of organ failure in ACLF patients [4] and frequently leading to the use of renal replacement therapies (RRT) [5].

Attaining sufficient antibiotic concentrations in critically ill patients is often cumbersome as these patients present with various factors influencing pharmacokinetics (PK): the volume of distribution (V_d_) may increase due to a capillary leak syndrome and presence of effusions as e. g., ascites, while antibiotic clearance (CL) may be decreased due to progression of organ dysfunction. Moreover, these processes are often dynamic which can lead to observed inter-occasion variability of these PK parameters.

As per linezolid labelling, no dose adaption is required for patients with liver cirrhosis Child–Pugh A and B, but no data are available for Child–Pugh C. Nevertheless, linezolid is partially metabolized, presumably by the cytochrome P450 system, but the total metabolic pathway is not fully understood, yet. Approximately 35% of linezolid are excreted renally and linezolid and its metabolites may be dialyzed [6, 7]. To investigate the effect of ACLF on the PK of linezolid in critically ill patients requiring RRT we compared this group with critically ill patients on RRT without ACLF.

## Methods

### Ethics

The study was approved by the Ethics Committee of the Hamburg Chamber of Physicians, Germany (Reference: PV5415). Consent was obtained from the patients’ closest relatives or legal representatives. The study was performed in accordance with the ethical standards as laid down in the 1964 Declaration of Helsinki and its later amendments or comparable ethical standards.

### Study design

The study was conducted as an open label observational prospective cohort study.

### Setting and population

The study was conducted in the Department of Intensive Care, University Medical Center, Hamburg-Eppendorf with twelve intensive care units (surgical, conservative, and interdisciplinary) and a total of 140 beds. Patients were eligible if they received linezolid for clinical indication and required continuous RRT. Patients < 18 years or with an extracorporeal circuit other than the RRT were excluded. According to liver function, patients were grouped into patients with ACLF and patients without ACLF (“no liver failure”, NLF).

ACLF was defined according to the definition of the Chronic Liver Failure (CLIF) consortium [4]. Presence of liver cirrhosis was diagnosed based on a combination of characteristic clinical (e.g., ascites, caput medusae, spider angiomata, etc.), laboratory and radiological findings (typical morphological changes of the liver, signs of portal hypertension, etc. in ultrasonography or computed tomography scanning), or via histology, if available [8].

### Medication

Linezolid (Dr. Friedrich Eberth Arzneimittel GmbH, Germany) was given over 30 min by infusion pump at a dose of 600 mg quid 12 h via a central venous line (short-term infusion).

### Renal replacement therapy

RRT was performed as continuous veno-venous hemodialysis (CVVHD) or as a postdilution continuous veno-venous hemofiltration (CVVH) as described before [9]. Both methods were performed with Multifiltrate pro^®^ dialysis machines using an Ultraflux^®^ AV1000S hollow-fiber hemofilter (Fresenius Medical Care, Bad Homburg, Germany) with a membrane surface area of 1.8 m^2^. For CVVHD, a regional citrate-calcium anticoagulation was used; and the targeted dialysate or replacement fluid dose was 30 mL/kg/h of actual body weight. CVVH was chosen in cases of severe acidosis due to the technically higher possible blood flow. No filter change occurred during the study period.

### Sampling and storage

Ultrafiltrate and pre- and postfilter blood samples were obtained at the following time points: T0 as the baseline before the first monitored infusion, 1 h (T1), 2 h (T2), 4 h (T4), 6 h (T6), 8 h (T8) and 12 h (T12) after the start of infusion. T12 was obtained before the next infusion of linezolid and served as a trough concentration. Furthermore, we obtained values after 24 h (before and 30 min after end of infusion, T24 and T25) and after 48 h (T48 and T49). In cases of CVVH, postfilter samples were obtained from the extracorporeal circuit before the addition of replacement fluid. All samples were centrifuged immediately, and the supernatant stored at − 20 °C until assayed.

### Assay

A well-established, validated, and accredited high performance liquid chromatography approach with diode array detection (HPLC/DAD), which has been used for routine meropenem, linezolid, piperacillin and ceftazidime analysis for more than 3 years was used for the analysis of linezolid in serum. Detailed information about the sample preparation and method settings have been previously described [10]. The method was used with the following slight modification. The assay was routinely calibrated using six linezolid standards of spiked blank human serum (0.8, 8, 12, 20, 30, 40 mg/L) with two independently prepared quality control samples (8 and 20 mg/L) included in each analytical series.

### Statistics

Microsoft Excel 2016 (Microsoft Corp., Redmond, WA, USA) was used for data management. The SPSS statistical software package (version 27, IBM Inc., Armonk, NY, USA) was used for descriptive statistical analysis. The pharmacokinetic analysis and Monte-Carlo simulation were performed with the non-linear mixed-effects modelling software NONMEM, version 7.4 (Icon Development Solutions, Ellicott City, MD, USA). Data are given as mean ± standard deviation or median and quartiles, as appropriate.

### Population pharmacokinetic analysis

The integrated dialysis pharmacometric (IDP) model was used to describe linezolid pharmacokinetics [11]. The IDP model allows for simultaneous analysis of RRT-related parameters as well as pre-filter (i.e., serum), post-filter and effluent concentration samples.

The RRT clearance for the pre- and post-filter concentrations in the IDP model is estimated as follows:

$${CL}_{RRT }={Q}_{blood\,adj}. \times \frac{{C}_{pre}-{C}_{post}}{{C}_{pre}}$$

$${Q}_{blood\,adj}.={Q}_{blood}\times \left(1-Hct+Hct\times \frac{{C}_{RBC}}{ {C}_{pre}}\right)$$

$${C}_{post,corr.}={C}_{post,meas.} \times \frac{{Q}_{blood\,adj. } - {Q}_{FRR}}{{Q}_{blood\,adj}.}$$

where the adjusted blood flow rate (*Q*_blood adj._) is being calculated using the blood flow (*Q*_blood_), the hematocrit (Hct) and the red blood cell-to-serum-ratio (*C*_RBC_/*C*_ppre_), with *C*_pre_ representing the pre-filter and *C*_post_ the post-filter serum concentration.

The RRT clearance for the effluent concentration in the IDP model is estimated as follows:

$${CL}_{RRT}={Q}_{effl.} \times \frac{{C}_{effl }}{{C}_{pre}}$$

$${Q}_{effl.}={Q}_{dial}+{Q}_{FRR}$$

with *Q*_effl._ representing the total effluent flow rate, *Q*_dial_ the dialysate flow rate, *Q*_FRR_ the fluid removal rate, and *C*_effl._ the concentration of drug in the effluent and *C*_pre_ the pre-filter serum concentration.

One- and two-compartment models with first-order elimination were evaluated to identify the model which best described linezolid concentration–time data. Between-subject variability (BSV) and between-occasion variability (BOV) of random effects were assumed to follow a log-normal distribution. The estimates of BSV and BOV were provided as percentage coefficient of variation (% CV). A combined additive and proportional error model was used to describe residual unexplained variability. First-order conditional estimation with eta-epsilon interaction (FOCE-I) was used to estimate population pharmacokinetic parameters of linezolid.

Model building was guided by the drop in the objective function value (ΔOFV; proportional to -2 log-likelihood), inspection of goodness-of-fit plots and overlay plots of individually predicted vs. observed PK measurements and visual predictive checks (VPC). A decrease in OFV of more than 3.84 between two nested models after the addition of one parameter was considered significant (corresponds to *p* < 0.05). Data visualization and evaluation of NONMEM output during the model development process were conducted with PsN 5.0.0 [12], and R (version 4.2.1, R Foundation for statistical computing, Vienna, Austria). The individual PK parameters were used to assess any potential differences between patients with ACLF and NLF patients. The differences in PK parameters between the two groups were tested by comparing the mean parameter values using the t-test.

### Simulations

The final model was used to perform Monte-Carlo simulations in 1000 virtual patients in presence or absence of ACLF receiving linezolid in the following doses, each given twice daily: 150 mg, 300 mg, 450 mg, 600 mg, and 900 mg. In the simulation, RRT flow rates of 120 ml/min, 150 ml/h and 2200 ml/h for blood, ultrafiltration, and dialysate were used, respectively. Day 2 trough concentrations were simulated and evaluated against the target range of 2–7 mg/L [13].

## Results

The linezolid serum concentration–time data used in this analysis were obtained in 18 critically ill patients on RRT, with 9 of these patients diagnosed with ACLF. A total of 334 serum concentrations and 120 effluent concentrations were available for analysis. Out of the 334 serum concentrations, 167 were pre-dialysis filter samples and 167 post-dialysis filter samples. The detailed patient demographics, and RRT modes and flow rates are depicted in Table 1. The flow rates differed between subjects and within subjects over the duration of the study. The linezolid measurements are given in the Additional file 1: Table S1.

**Table 1:** Clinical characteristics of patients

	NLF	ACLF	Combined
Number of patients	9	9	18
Males	5 (56%)	7 (78%)	
Number of PK samples	259	195	454
Number of PK samples per patient	30 (12–33)	21 (12–31)	25 (12–33)
Age (Years)	69 (8)	52 (13)	61 (14)
Weight (kg)	88 (15)	87 (11)	87 (13)
Height (cm)	172 (7)	177 (8)	
BMI (kg/m^2^)	29.7 (4.8)	27.8 (4.5)	
APACHE II	28 (10)	30 (10)	29 (10)
SAPS II	52 (15)	56 (14)	54 (14)
MELD	n/a	31 (7)	n/a
CLIF-SOFA	n/a	15 (4)	
PT (%)	83 (21)	60 (28)	72 (27)
Bilirubin (mg/dL)	2.0 (3.0)	10.8 (10.7)	6.4 (8.9)
Antithrombin (%)	85 (25)	34 (22)	59 (35)
RRT modes and flow rates			
CVVHD (*n*)	8	5	
Blood flow (ml/min)	100 (80–120)	120 (100–350)	
Dialysate flow (ml/h)	2000 (1500–3300)	2400 (2000–4800)	
Ultrafiltration (ml/h)	150 (0–250)	150 (0–200)	
CVVH (*n*)	1	4	
Blood flow (ml/min)	200	200 (150–200)	
Replacement fluid (ml/h)	2000	2000 (2000–4000)	
Ultrafiltration (ml/h)	150	0 (0–80)	

*NLF* patients without liver failure, *ACLF* acute-on-chronic liver failure due to liver cirrhosis, *BMI* Body Mass Index, *APACHE II* Acute Physiology and Chronic Health Evaluation, *SAPS II* Simplified Acute Physiology Score, *MELD* Model of End Stage Liver Disease, *CLIF-SOFA* Chronic Liver Failure Consortium Sequential Organ Failure Assessment, *PT* prothrombin time, *RRT* renal replacement therapy, RRT flow rates at time point T0, data are given as mean (standard deviation) or median (range)

### Structural model and parameter estimates

**Fig. 1:**
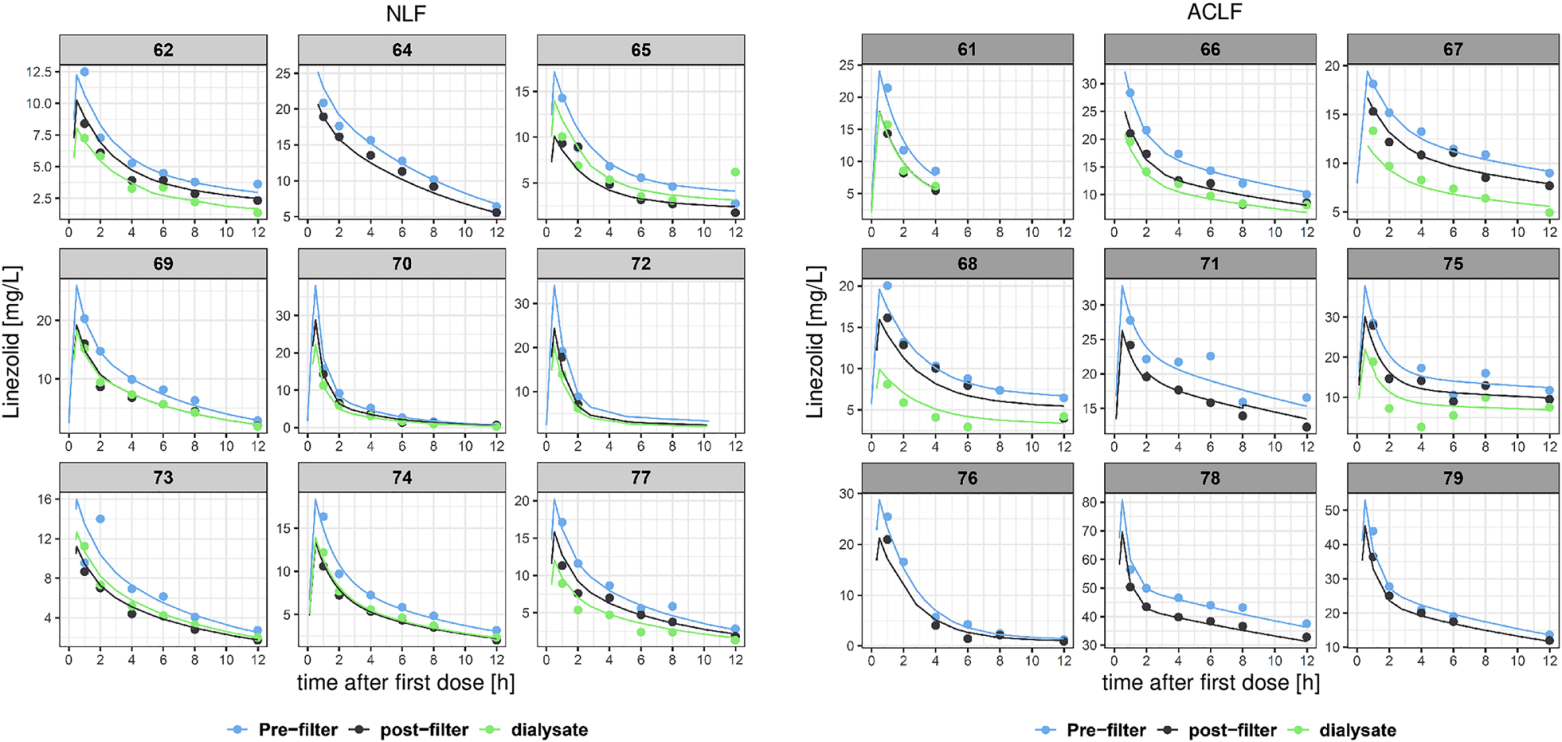
Individual predicted linezolid concentrations versus time after the first dose (0–12 h). The blue line represents the pre-filter serum concentrations, the black line represents the post-filter serum concentrations, the green line represents the effluent concentrations, and the dots are the observed concentrations. *NLF* no liver failure, *ACLF* acute-on-chronic liver failure

**Fig. 2:**
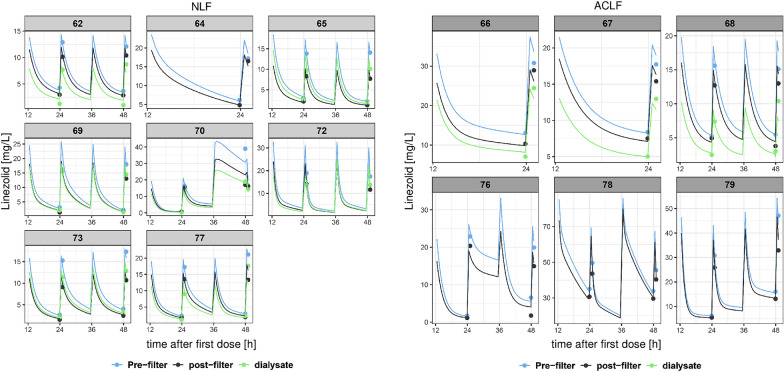
Individual predicted linezolid concentrations versus time after the first dose (12–50 h) on a normal scale. The blue line represents the pre-filter serum concentrations, the black line represents the post-filter serum concentrations, the green line represents the effluent concentrations, and the dots are the observed concentrations. *NLF* no liver failure, *ACLF* acute-on-chronic liver failure

**Fig. 3:**
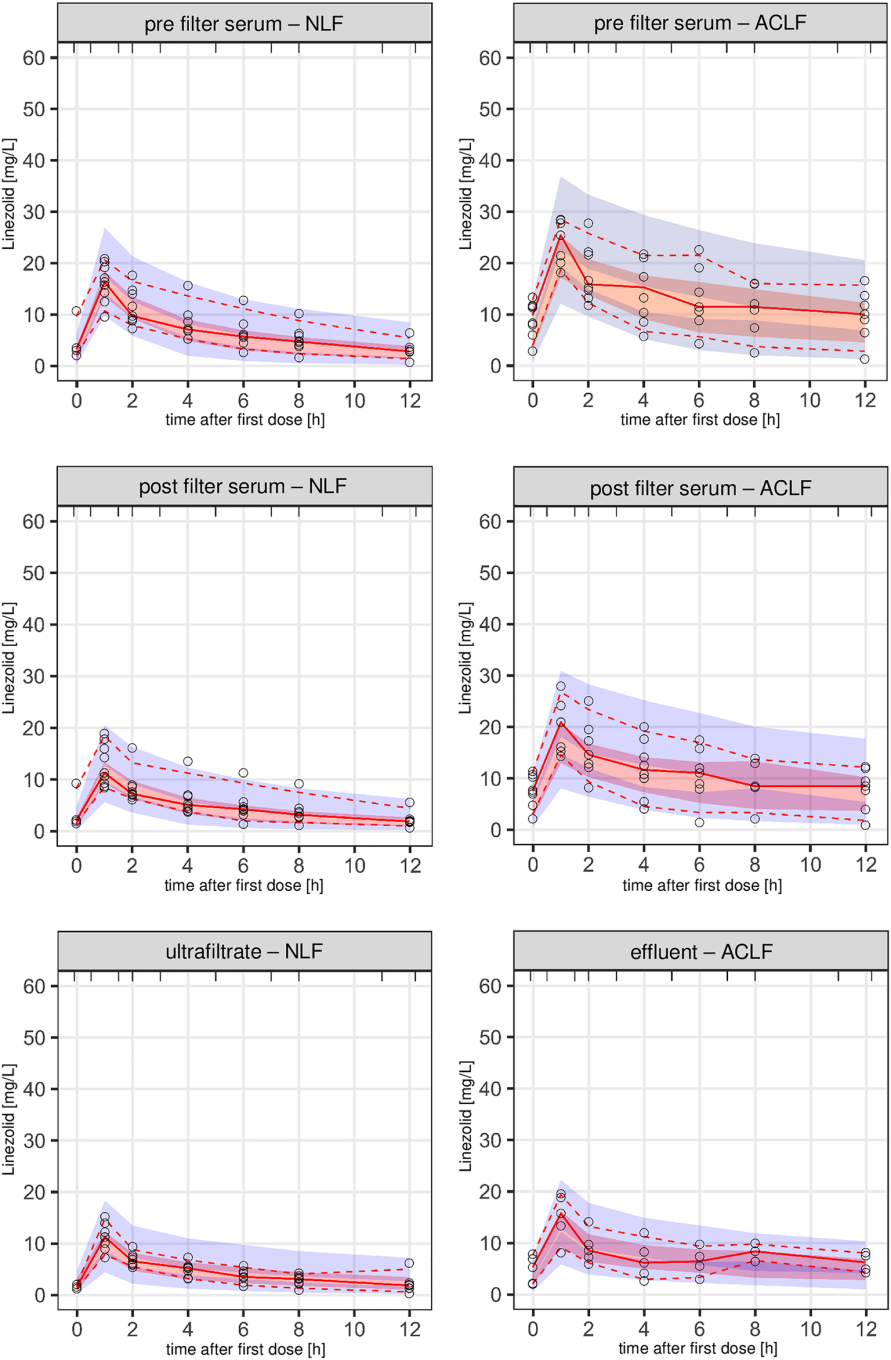
Prediction-corrected visual predictive check of linezolid concentrations versus time after the first dose. Stratified by serum and effluent concentrations between groups. The solid and dashed lines represent the 5th, 50th, and 95th percentiles of the observed data, while the shaded areas represent the model-predicted 95% confidence intervals for the same percentiles. The dots are the observed concentrations. *NLF* no liver failure, *ACLF* acute-on-chronic liver failure

Linezolid serum PK was best described by a two-compartment model with first-order elimination. The estimated typical linezolid body clearance for a patient without liver disease was 6.17 L/h while RRT clearance was 1.65 L/h. Individual predicted linezolid concentrations are shown in Figs. 1, 2. No adsorption to the membrane was estimable. The final parameter estimates are presented in Table 2 and a VPC showing suitable fit is shown in Fig. 3.

**Table 2:** Parameter estimates of the final model of linezolid

Model parameter		Typical value		Variability
	Value	95% CI	% CV	95% CI
Body clearance (L/h)	6.17	4.97; 7.55	11.6 (BSV) 60.3 (BOV)	1.43; 28.6 45.9; 80.5
Central volume of distribution (L)	27.5	22.3; 33.3	43.0 (BSV) 6.60 (BOV)	30.33; 62.5 0.89; 18.7
RRT clearance (L/h)	1.65	1.39; 1.94	33.8 (BSV)	23.4; 46.6
Inter-compartmental clearance (L/h)	9.58	7.97; 11.2		
Peripheral volume of distribution (L)	43.7	22.3; 77.1	104.9 (BSV) 112.7 (BOV)	87.2; 142
Change in body clearance for patients with liver disease (%)	− 78.7	− 87.0; − 67.1		
Pre-filter proportional error (%)	12.0	10.4; 14.1		
Post-filter proportional error (%)	14.9	12.7; 16.7		
Dialysate proportional error (%)	22.9	19.8; 26.8		

*BSV* Between-subject variability, *BOV* between-occasion variability, *RRT* renal replacement therapy, *95% CI* 95% confidence interval, *% CV* Percent Coefficient of Variation

Patients with ACLF exhibited a body clearance of 1.32 L/h, corresponding to a 79% reduction in body clearance when compared to patients without ACLF (ΔOFV = − 12.4, *p* < 10^–4^). This was further confirmed by an analysis of the empirical Bayesian estimates of the individual PK parameters using the *t*-test which found body clearance as the most significantly different PK parameter between the two groups. The results are presented in Table 3.

**Table 3:** Comparison of individual pharmacokinetic parameter estimates of patients with and without ACLF

	NLF	ACLF	*p*-value
Number of patients	9	9	n/a
Body clearance (L/h)	6.26 (2.43)	1.54 (0.52)	< 0.001
Central volume of distribution (L)	34.0 (9.04)	26.3 (11.3)	< 0.001
RRT clearance (L/h)	1.58 (0.403)	1.78 (0.61)	0.203
Peripheral volume of distribution (L)	122 (172)	110 (161)	0.463
Inter-compartmental clearance (L/h)	9.58(0)	9.58 (0)	n/a

*NLF* no liver failure, *ACLF* acute-on-chronic liver failure, *RRT* renal replacement therapy, parameters are reported as mean (sd), *n/a* not applicable

### Simulation

**Fig. 4:**
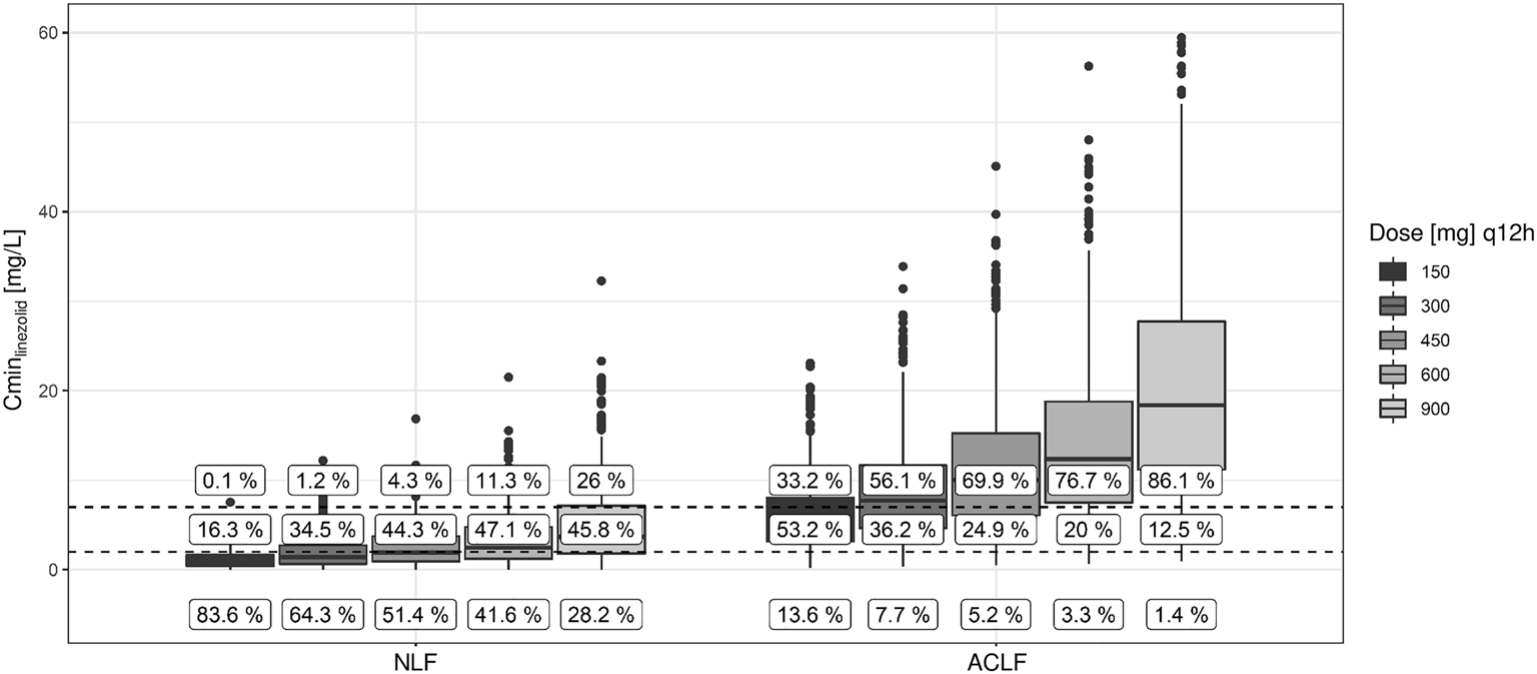
Target attainment. Target attainment (labels) for minimum linezolid concentration (Cmin) between 2 and 7 mg/L (dashed lines) calculated using Monte Carlo Simulations with the developed pharmacometric model using twice daily dosing of 150–900 mg. *NLF* no liver failure, *ACLF* acute-on-chronic liver failure

The probability to attain the target of a trough concentration of 2–7 mg/L for patients receiving the standard linezolid dose of 600 mg twice daily in the group without liver disease was 47%, while 42% were underexposed and 11% were overexposed. In the ACLF group, 77% of the simulated patients were overexposed, 20% were in the target range, and only 3% were underexposed. The distribution of the trough concentration values is presented in Fig. 4. The highest probability of exposure in the recommended range of 2–7 mg/L was attained in the NLF group at the standard dose of 600 mg twice daily and at 150 mg twice daily in the ACLF group.

## Discussion

In this study, we used the IDP model to characterize the PK of linezolid in critically ill patients undergoing RRT and assessed the impact of ACLF on PK and PTA of linezolid [11]. The model identified a 79% reduction in linezolid body clearance in patients with ACLF. The decreased linezolid clearance in patients with ACLF is similar to the decreased linezolid clearance reported in patients with severe liver cirrhosis (50%) [14] and in patients after liver transplantation (60%) [15]. In patients without organ dysfunction, 35% of linezolid are excreted renally, 50% are metabolized into the two major inactive metabolites PNU-142586 and PNU-142300, and 10% are excreted with the feces [7]. The metabolism is unrelated to the typical cytochrome P450 isoenzymes 1A2, 2C9, 2C19, 2D6, 2E1, and 3A4 that play a major role in drug interactions [16] and therefore, linezolid metabolism was believed to be entirely unrelated to the cytochrome system. However, recent data indicate that the two cytochrome isoenzymes CYP2J2 and CYP4F2 metabolize linezolid [17] which may be influenced by liver failure and thus could explain the reduced body clearance in ACLF as shown in our study. Linezolid and its main metabolites may be removed by hemodialysis and a clearance of 1.88 L/h by CVVH has been determined in critically ill patients which is similar to our results [18]. A systematic review found clearances of 1.2–2.3 L/h for CVVH, 0.9–2.2 L/h for CVVHDF and 2.3 L/h for CVVHD while highlighting a wide variability of PK parameters [19]. As in our study, the observed linezolid population PK parameters were highly variable. Total clearance and volume of distribution values varied widely. Our IDP model did not detect any adsorption of linezolid to the hemofilter membrane. This finding is supported by previous experimental data, showing no absorption to hemofilter membranes [20] or extracorporeal membrane oxygenators [21].

Concerning the mechanism of action, linezolid binds to the 50S subunit of the prokaryotic ribosome, preventing formation of the initiation complex for protein synthesis [22]. Under clinical circumstances, linezolid exhibits only gram-positive activity due to efflux pumps prevalent in most gram-negative bacteria [22]. Time-kill curves indicate a bacteriostatic effect against *S. aureus* and *Enterococcus spp.* [23] which may explain data suggesting an inferior clinical efficacy compared to beta-lactams or glycopeptides [24]. However, the latter also may be explained by insufficient target attainment because no TDM was performed in that study. Moreover, inconsistent clinical targets with the following clinical efficacy PK/PD targets have been proposed [13, 25, 26]: a trough concentration of 2–7 mg/L, an AUC_0–24 h_/MIC of 80–120 mg*h and a time above MIC of ≥ 85%. Trough concentrations above 7 mg/L have been linked to an increase of toxicity, thus making precise targeting particularly important for linezolid. In our study, we evaluated the appropriateness of the standard dosing regimen of 600 mg twice daily based on the developed pharmacometric model with regard to attainment of a trough concentration range of 2–7 mg/L [13]. The most marked finding was that 77% of the simulated patients with liver disease were overexposed. This could also be demonstrated in a very recent clinical trial showing a significantly higher risk for overexposure in patients with severe liver failure [27]. Too high concentrations of linezolid have been associated with thrombocytopenia [28]. Linezolid use in patients with liver failure has also been previously linked to thrombocytopenia, but no TDM was performed in this study and we suggest that the higher incidence of thrombocytopenia may be explained by undetected linezolid overexposure [29]. Furthermore, mitochondrial ribosomal inhibition may lead to lactic acidosis [30, 31], although prolonged therapy and not the peak concentration may be the main risk factor [32]. Interestingly, even in our group without liver disease, solely 47% of the patients were in the target range with approximately 40% being underdosed. These cases are prone to therapy failure and would require an increase of the linezolid dose.

The V_d_ was lower in the ACLF group. We cannot give a conclusive explanation for this finding as protein binding of LZD is only 31% and should not relevantly influence the V_d_. Ascites which is often present in ACLF patients may increase and not decrease V_d_ as has been shown for meropenem [10]. However, the lower V_d_ may be a contributing factor to the more frequent overexposure to linezolid in the ACLF group. Due to this lower V_d_ and in connection with the reduced body clearance as outlined above, a reduction of the linezolid dose in ACLF patients seems reasonable before TDM results are available. Because the V_d_ and the reduced body clearance are independent of RRT, we suggest that a dose reduction should also be considered in ACLF patients that do not receive RRT.

Our study has the following limitations. The sample size included only 9 patients per groups, but this is a typical number of patients in PK studies [9, 10, 33, 34]. We did not systematically evaluate thrombocytopenia or lactic acidosis as common adverse effects of linezolid because both findings are typical in the included population of critically ill patients, which prevents correct causal attribution. We included different modes of continuous RRT with more patients in the ACLF group receiving CVVH, but our results from the IDP model indicate that type of RRT did not have an influence on the linezolid clearance.

## Conclusion

We developed a PK model for linezolid in critically ill patients receiving continuous RRT with and without ACLF. Linezolid body clearance in patients with ACLF was markedly lower compared to patients without liver failure while the central volume of distribution was slightly decreased. Given the overall high variability in the present cohort, TDM with dose adjustments seems required to reach target attainment. Until results from TDM are available, a reduction of linezolid dose may be considered in ACLF patients to prevent overdosing due to the low body clearance and lower V_d_.

### Supplementary Information

**Additional file 1: Table S1.** Linezolid Concentrations at Time Points.

## Data Availability

The data are available from the corresponding author upon reasonable request.
